# Microbiota Analysis for the Optimization of *Campylobacter* Isolation From Chicken Carcasses Using Selective Media

**DOI:** 10.3389/fmicb.2019.01381

**Published:** 2019-06-21

**Authors:** Jinshil Kim, Hakdong Shin, Hyeeun Park, Hayan Jung, Junhyung Kim, Seongbeom Cho, Sangryeol Ryu, Byeonghwa Jeon

**Affiliations:** ^1^Department of Food and Animal Biotechnology, Department of Agricultural Biotechnology, Center for Food and Bioconvergence, Research Institute for Agriculture and Life Sciences, Seoul National University, Seoul, South Korea; ^2^Department of Food Science and Biotechnology, College of Life Science, Sejong University, Seoul, South Korea; ^3^College of Veterinary Medicine and Research Institute for Veterinary Science, Seoul National University, Seoul, South Korea

**Keywords:** microbiota compositions, selective enrichment, *Campylobacter*, 16S rRNA sequencing, bacterial isolation

## Abstract

Since contaminated poultry meat is the major source of transmitting *Campylobacter jejuni* to humans, the isolation of *Campylobacter* from poultry carcasses is frequently performed in many countries as a baseline survey to ensure food safety. However, existing isolation methods have technical limitations in isolating this fastidious bacterium, such as a growth competition with indigenous bacteria in food samples. In this study, we compared the differences in microbiota compositions between Bolton and Preston selective media, two most common selective media to isolate *Campylobacter*, and investigated how different microbiota compositions resulting from different enrichment methods may affect isolation frequencies. A next-generation sequencing (NGS) analysis of 16S *rRNA* demonstrated that Bolton and Preston-selective enrichments generated different microbiota structures that shared only 31.57% of Operating Taxonomic Unit (OTU) types. Particularly, *Escherichia* was highly prevalent in Bolton selective media, and the enrichment cultures that increase *Escherichia* negatively affected the efficacy of *Campylobacte*r isolation. Furthermore, the combination of the selective media made a significant difference in the isolation frequency. The Bolton broth and Preston agar combination exhibited the highest (60.0%) frequencies of *Campylobacter* isolation, whereas the Bolton broth and Bolton agar combination showed the lowest (2.5%). These results show that each selective medium generates a unique microbiota structure and that the sequence of combining the selective media also critically affects the isolation frequency by altering microbiota compositions. In this study, we demonstrated how a microbiota analysis using NGS can be utilized to optimize a protocol for bacterial isolation from food samples.

## Introduction

*Campylobacter* spp., particularly *Campylobacter jejuni*, is a leading bacterial cause of gastroenteritis worldwide (Kirk et al., 2015). As a post-infection complication, *C. jejuni* is also implicated in the development of Guillain-Barré syndrome (GBS), an acute flaccid paralysis, accounting for approximately 31% of GBS cases (Poropatich et al., 2010). As poultry is the primary host for *Campylobacter*, human campylobacteriosis is frequently caused by the consumption of contaminated poultry meat (Newell and Fearnley, 2003; Rosenquist et al., 2003). Despite the well-established commensalism between *Campylobacter* and poultry, interestingly, *Campylobacter* prevalence in poultry highly varies depending on the country; for example, 27.1% in the Netherlands (van de Giessen et al., 2006), 38.6% in Poland (Szczepanska et al., 2017), 72.9% in the UK (Kaakoush et al., 2015), and 80.9% in Cambodia (Lay et al., 2011). Although the variations may result from various factors, such as geographical differences, sampling seasons, and food product types (Sahin et al., 2015), different isolation procedures used in different laboratory settings (e.g., different selective media and enrichment methods) may also significantly affect the isolation of this fastidious bacterium (Levesque et al., 2011; Carrillo et al., 2014).

The Bolton *Campylobacter*-selective supplement, the Preston *Campylobacter*-selective supplement, and modified charcoal-cefoperazone-deoxycholate agar (mCCDA) media are frequently used to isolate *Campylobacter* from food samples (Corry et al., 1995; Bojanić et al., 2017; Narvaez-Bravo et al., 2017; Vinueza-Burgos et al., 2017). The selective enrichment of *Campylobacter* is based on the intrinsic resistance of *Campylobacter* to antimicrobials. For instance, the antibacterial component of mCCDA is cefoperazone, the Bolton *Campylobacter*-selective supplement contains three antibiotics (cefoperazone, vancomycin, and trimethoprim), and the Preston *Campylobacter*-selective supplement has three antibiotics (polymyxin B, rifampicin, and trimethoprim), and *Campylobacter* is intrinsically resistant to these antibiotics (Taylor and Courvalin, 1988; Corry et al., 1995). The International Organization for Standardization (ISO) protocol for *Campylobacter* isolation employs the enrichment with Bolton *Campylobacter*-selective broth and subsequent culture on mCCDA (ISO 10272: 2006). However, the high prevalence of antibiotic-resistant bacteria among the microbiota of poultry carcasses has significantly compromised the effectiveness of *Campylobacter* isolation using the selective media. Particularly, extended-spectrum beta-lactamase (ESBL)-producing *Escherichia coli*, which is resistant to cephalosporins, is highly prevalent in poultry (Overdevest et al., 2011). ESBL *E. coli* can grow on the Bolton selective media and mCCDA, both of which contain cefoperazone, a third-generation cephalosporin antibiotic (Hazeleger et al., 2016). Although microbiota compositions after *Campylobacter* enrichment with selective media may significantly impact the isolation frequency of *Campylobacter*, nothing is known about how different microbiota compositions may affect the efficacy of *Campylobacter* isolation.

Next-generation sequencing (NGS) technology allows for the sequencing of massive samples and is useful for the microbiota analysis (Lee et al., 2013; Bragg and Tyson, 2014). By using NGS, in this study, we investigated microbiota compositions in chicken carcasses during the selective enrichment of *Campylobacter* with Bolton and Preston selective media, two selective media most frequently used to isolate *Campylobacter*. Based on the microbiota compositions, furthermore, we optimized the procedures of *Campylobacter* isolation using the two selective media. To the best of our knowledge, this is the first study to optimize a bacterial isolation protocol based on a microbiota analysis.

## Materials and Methods

### Sample Collection and Selective Enrichment

Forty whole chicken carcass samples from 23 different brands were purchased from retail stores in South Korea from March 31 to July 21, 2017. Raw whole chicken samples were selected based on the production date and the shelf-life, packaged in a polyethylene bag, and delivered to the laboratory on ice. A whole chicken carcass was divided in half and subjected to enrichment with 1 L of Bolton broth with Bolton *Campylobacter*-selective supplements (Oxoid, UK) and Preston broth with Preston *Campylobacter*-selective supplements (Thermo-Fisher Scientific, USA) in plastic bags (Ziploc^®^, SC Johnson Co.). Although mCCDA is frequently used to isolate *Campylobacter*, in this study, Bolton and Preston selective media were used because the antimicrobial component of mCCDA (i.e., cefoperazone) is also present in Bolton. Samples were not rinsed with peptone water not to cause microbiota changes. The bags were incubated under microaerobic conditions (4% H_2_, 6% O_2_, 7% CO_2_, 83% N_2_) at 42°C for 24 h.

### DNA Extraction and 16S rRNA Amplification and Sequencing

Twenty chicken samples from 19 different brands and products were used to compare the differences in microbiota compositions after enrichment with Bolton and Preston selective media as mentioned above. Total DNA was extracted from 300 μl of each enrichment culture using a commercial DNA extraction kit (FastDNA SPIN™ kit for soil, MP Biomedical, Santa Anna, CA) according to the manufacturer’s instructions. DNA was eluted in 100 μl FastDNA elution buffer, and the extracted DNA was quantified by NanoVue Plus spectrophotometry before dilution to 15 ng/μl. The V3/V4 region of the 16S rRNA gene was amplified using the universal primers 341F and 805R on the following PCR reaction conditions (95°C for 5 min; 30 cycles at 95°C for 30 s, 55°C for 30 s, 72°C for 30 s; 72°C for 7 min). PCR products were purified using a High Pure PCR Product Purification Kit (Roche Applied Science, Germany). Paired-end (2 × 301 bp) sequencing was performed commercially (Chunlab Inc., Seoul, Korea) using a MiSeq platform (Illumina, San Diego, USA).

### 16S rRNA Gene-Based Sequencing Analysis

The 16S rRNA gene sequences were analyzed using Quantitative Insights Into Microbial Ecology (QIIME) software package (v1.9.1) (Caporaso et al., 2010). Quality filtered sequences (Phred ≥ Q20) were used for identifying operational taxonomic units (OTUs) with open-reference OTU picking method in accordance with 97% identity of EzTaxon database (v1.5) (Yoon et al., 2017). Chimeric sequences were removed by UCHIME (Edgar et al., 2011). All samples were rarefied to 15,030 reads per samples for bacterial diversity analysis. To evaluate alpha diversity (microbial diversity within samples) of samples, alpha rarefaction was plotted using the phylogenetic distance and the detected number of species metrics with 10 iterations. The unweighted and weighted UniFrac distances were calculated for beta diversity analysis (Lozupone et al., 2006), and PERMANOVA was used to test the dissimilarity of beta diversity between groups (Anderson, 2001). Paired *t*-test was used for the statistical analysis of paired sample sets. A non-parametric *t*-test was performed with 10,000 permutations to test significance in intra-group distances. Linear discriminant analysis effect size (LEfSe) (Segata et al., 2011) was used to identify significant differences (LDA score > 3.0) in the relative abundance of bacterial taxonomy.

### *Campylobacter* Isolation Frequencies of the Four Media Combinations

The enrichment cultures (20 ml) were concentrated by centrifugation at 4,000×*g*, 4°C for 7 min, and pellets were resuspended with 1 ml of each supplement broth (Bolton broth or Preston broth) (Son et al., 2007). After 10-fold serial dilution with PBS, bacterial suspension (100 μl) was spread onto Bolton and Preston agars containing each *Campylobacter*-specific supplements. Four different media combinations were made in the study, including Bolton broth-Bolton agar (BB-BA), Bolton broth-Preston agar (BB-PA), Preston broth-Bolton agar (PB-BA), and Preston broth-Preston agar (PB-PA). Inoculated agar plates were incubated microaerobically at 42°C for 48 h. Based on the colony morphology, such as flat, shiny, and mucoid colonies, 10 presumptive *Campylobacter* spp. colonies were confirmed by multiplex PCR using primer sets for *Campylobacter*-specific 16S rRNA gene and four *Campylobacter* species-specific primers were used; the primer sequences were described in Supplementary Table S3. All *Campylobacter* isolates were grown on Mueller-Hinton (MH) agar at 42°C under microaerobic conditions and stored in MH broth with 15% glycerol at −81°C.

### Taxonomical Identification of Colonies Growing on *Campylobacter*-Selective Agars

To compare the distribution of *Campylobacter* and non-*Campylobacter* strains in BB-PA and PB-BA, 15 colonies were randomly selected from a *Campylobacter*-selective agar in each media combination based on colony shape, color, size, and transparency and transferred to a fresh *Campylobacter*-selective agar plate for pure culture. After 1–2 days incubation, colonies were picked up and boiled at 95°C for 7 min, and the boiled supernatant was used for PCR amplification of 16S rRNA gene. The PCR amplicons were purified and commercially sequenced (Macrogen, Inc., South Korea) by 3730xl DNA analyzer (Thermo Fisher Scientific), and the results were analyzed using BLASTN with 16S rRNA gene database.

## Results

### Microbiota Compositions in Chicken Carcasses After Enrichment With Bolton and Preston *Campylobacter*-Selective Media

**Figure 1:**
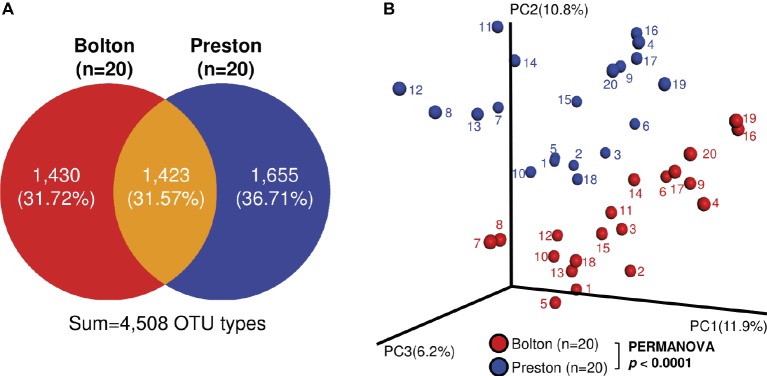
Bacterial beta diversity in the chicken samples enriched with Bolton and Preston *Campylobacter*-selective media. **(A)** Venn diagram showing detected OTU types. **(B)** Principal Coordinate Analysis (PCoA) plot of bacterial communities in chicken samples enriched with Bolton and Preston selective media. Unweighted UniFrac distances were used to evaluate diversity between samples, and PERMANOVA was used to test the dissimilarity of bacterial population structures.

In the 40 enrichment samples (20 samples per each selective culture), we obtained 1,741,595 sequences (paired-end, Phred ≥Q20) with an average of 43,540 reads per sample and binned into 4,508 operating taxonomic units (OTUs) (Supplementary Table S1). Although the bacterial alpha diversity did not show any significant differences between BB and PB (Supplementary Figure S1), only 31.57% (1,423) of OTU types were shared in the samples enriched with BB and PB (Figure 1A). The microbiota compositions after the selective enrichment with BB and PB were significantly different in bacterial structures (Figure 1B). Even though the same chicken carcass was divided and exposed to the two different selective enrichment conditions, there was a significant tendency to cluster depending on the type of selective media, not the sample (Figure 1B).

**Figure 2:**
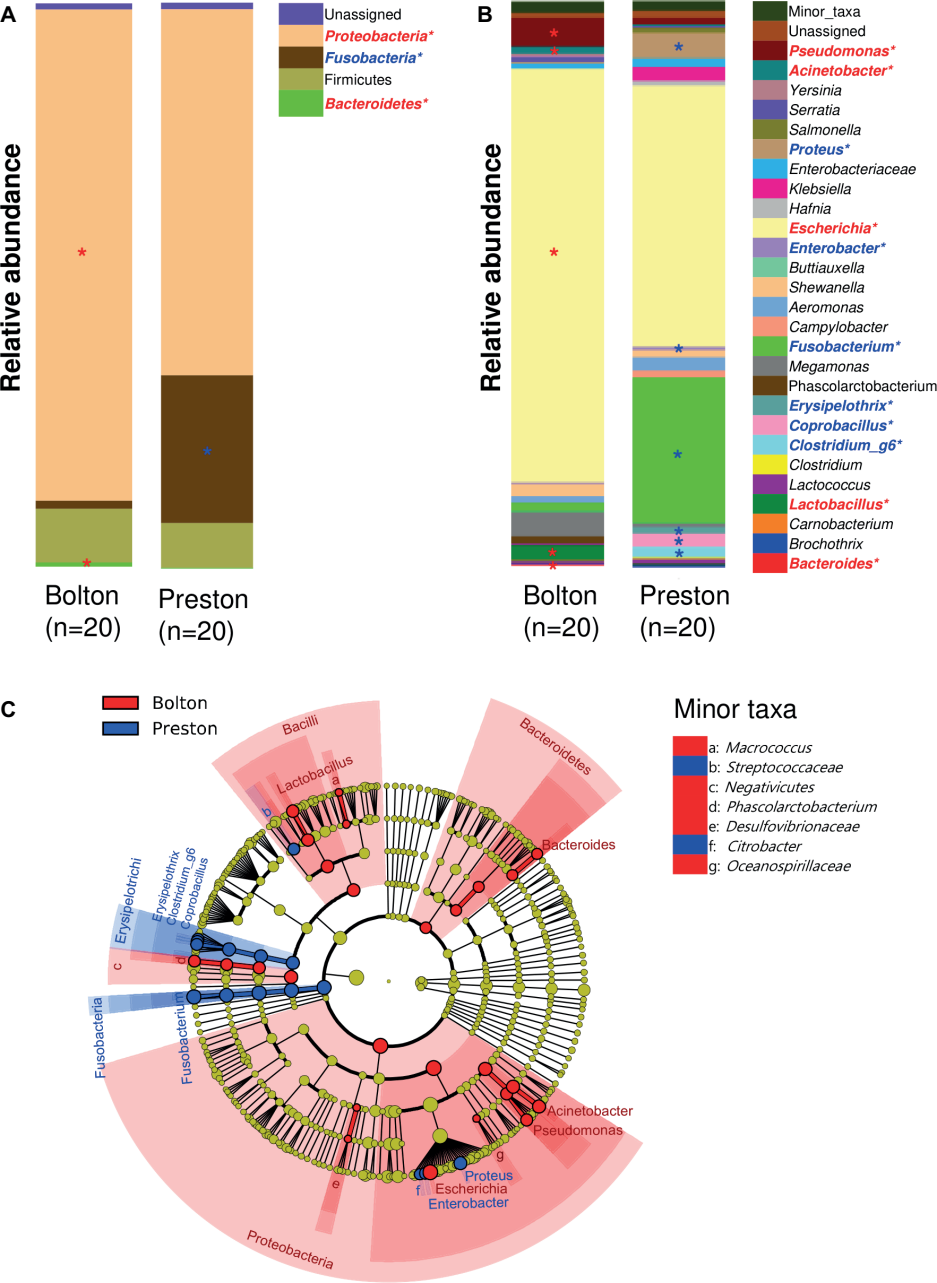
Comparisons of bacterial taxa in chicken samples enriched with Bolton and Preston *Campylobacter*-selective media. **(A,B)** Bacterial taxonomy at the levels of phylum **(A)** and genus (top 27 bacterial taxonomies) **(B)** is indicated by a different color. *Overrepresented taxa (with LDA > 3.0) in comparison with the other selective medium (Red, Bolton media; Blue, Preston media). **(C)** Cladogram of overrepresented taxa (with LDA > 3.0) in each group. Overrepresented taxa in Bolton and Preston media were indicated in red and blue, respectively.

**Figure 3:**
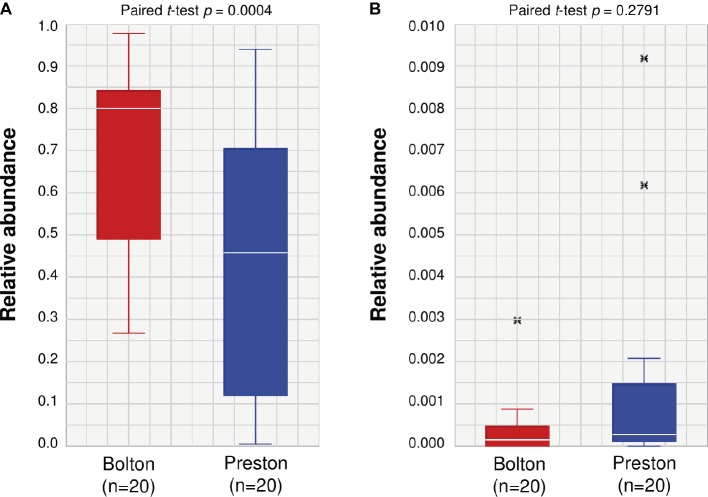
Relative abundance of *Escherichia* **(A)** and *Campylobacter* **(B)** in chicken samples enriched by Bolton and Preston *Campylobacter*-selective media. The paired *t*-test is used to compare two population means. x: outlier samples.

Proteobacteria and Firmicutes were the major phyla after the incubation of chicken samples in BB, whereas Proteobacteria and Fusobacteria were dominant after the PB enrichment (Figure 2A). At the genus level, the BB resulted in the enrichment of *Pseudomonas* (6.7%), *Acinetobacter* (1.4%), *Escherichia* (70.6%), *Phascolarctobacterium* (1.3%), *Lactobacillus* (2.5%), and *Bacteroides* (0.4%), whereas PB significantly enriched *Proteus* (5.1%), *Enterobacter* (0.6%), *Fusobacterium* (24.8%), *Erysipelothrix* (1.2%), *Coprobacillus* (2.6%), and *Clostridium_g6* (1.9%) (Figures 2B,C, Table 1). Notably, *Escherichia* was predominant in both BB and PB. Especially, the samples enriched by BB (70.6%) showed a higher abundance of *Escherichia* than PB (43.6%) (Figure 3A, Table 1). The PB enrichment exhibited a slightly higher abundance of *Campylobacter* compared to the BB enrichment, but the difference was not statistically significant (Figure 3B). The BB-enriched group exhibited less inter-individual differences than the PB-enriched group, indicating that the microbiota of chicken carcasses after the BB enrichment were more similar to each other compared to those generated by the PB enrichment (Supplementary Figure S2).

**Table 1:** Relative abundance of overrepresented bacterial taxonomies (LDA > 3.0) in Bolton and Preston *Campylobacter*-selective media.

Bacterial taxonomy	Bolton broth			Preston broth		
	Average (%)	SD (%)	LDA score	Average (%)	SD (%)	LDA score
*Pseudomonas*	0.066993	0.107706	4.349024	0.018384	0.044282	–
*Acinetobacter*	0.01428	0.024536	3.796018	0.002345	0.005418	–
*Escherichia*	0.706185	0.205485	5.131531	0.435565	0.296455	–
*Phascolarctobacterium*	0.012729	0.030239	3.806587	0.000315	0.001248	–
*Lactobacillus*	0.025155	0.033968	4.037203	0.002922	0.011788	–
*Bacteroides*	0.003504	0.00653	3.260713	0.000136	0.000323	–
*Proteus*	0.001982	0.003262	–	0.051391	0.113173	4.363844
*Enterobacter*	0.001137	0.002599	–	0.006445	0.012079	3.458029
*Fusobacterium*	0.024574	0.101321	–	0.248409	0.322747	5.063090
*Erysipelothrix*	0	0	–	0.011613	0.038189	3.654410
*Coprobacillus*	0.000047	0.000109	–	0.026078	0.070242	4.091141
*Clostridium_g6*	0.00001	0.000032	–	0.018655	0.037892	3.987605

### Effects of the Second Selective Cultivation on the Efficacy of *Campylobacter* Isolation

For *Campylobacter* isolation, it is a common procedure to transfer the first selective enrichment culture to the second selective agar media to maximize isolation rates. To increase the selective pressure in *Campylobacter* isolation, an aliquot of each selective enrichment broth was transferred to selective agars containing the other selective supplement. For instance, an aliquot from the BB enrichment culture was transferred to Preston *Campylobacter*-selective agar (PA). Although *Escherichia* was predominantly (70.6%) enriched in BB (Figure 3), the prevalence of *Escherichia* was reduced to 37.8% after transfer to PA (Table 2). Although *Escherichia* was relatively lower (43.5%) in PB compared to BB (Figure 3), *E. coli* became predominant (84.3%) when transferred to Bolton *Campylobacter*-selective agar (BA) (Table 2). Interestingly, the proportions of *Campylobacter* spp., including *C. jejuni* and *C. coli*, increased to 37.5% (*C. jejuni* 36.7% and *C. coli* 0.8%), when BB was transferred to PA (Table 2). Whenever the Bolton selective supplement was used, *E. coli* became predominant, and the proportion of *Campylobacter* was reduced (Table 2, Figure 3). The frequency of *Campylobacter* isolation was only 6.3% in Preston broth-Bolton agar (PB-BA) (Table 2). Based on the results, the Preston selective supplement was relatively effective in reducing *E. coli* and increasing *Campylobacter* (Figure 3, Tables 1, 2). Although the same selective media (Bolton and Preston) were used, interestingly, the sequence of combining the selective media made a significant difference in the frequencies of *Campylobacter* isolation (Table 2). The results showed that both the type of selective media and the sequence of combining selective media may significantly affect the frequencies of *Campylobacter* isolation by changing microbiota composition.

**Table 2:** Proportions of bacterial species isolated with Bolton broth-Preston agar (BB-PA) and Preston broth-Bolton agar (PB-BA) combinations.

Bacterial species	BB-PA	PB-BA
*Citrobacter amalonaticus*	1 (0.2%)	0 (0.0%)
*Escherichia coli*	227 (37.8%)	506 (84.3%)
*Escherichia albertii*	0 (0.0%)	2 (0.3%)
*Enterobacteriaceae bacterium*	1 (0.2%)	0 (0.0%)
*Erysipelotrichaceae bacterium*	3 (0.5%)	0 (0.0%)
*Erwinia* sp.	0 (0.0%)	1 (0.2%)
*Klebsiella pneumoniae*	15 (2.5%)	17 (2.8%)
*Fusobacterium mortiferum*	0 (0.0%)	3 (0.5%)
*Lactobacillus crispatus*	1 (0.2%)	0 (0.0%)
*Lactobacillus salivarius*	1 (0.2%)	0 (0.0%)
*Lactococcus lactis*	2 (0.3%)	1 (0.2%)
*Proteus mirabilis*	91 (19.6%)	27 (4.5%)
*Salmonella enterica*	9 (1.5%)	4 (0.7%)
*Staphylococcus aureus*	0 (0.0%)	1 (0.3%)
*Campylobacter coli*	5 (0.8%)	0 (0.0%)
*Campylobacter jejuni*	220 (36.7%)	38 (6.3%)
**Total number of isolates**	**600 (100%)**	**600 (100%)**

### Effects of the Sequence of Selective Medium Combinations on *Campylobacter* Isolation

To determine how the sequence of combining Bolton and Preston selective media affects the frequencies of *Campylobacter* isolation, we determined the isolation frequencies of four different combinations, including Bolton broth-Bolton agar (BB-BA), BB-PA, Preston broth-Preston agar (PB-PA), and PB-BA, using 40 retail chicken samples. Interestingly, each combination exhibited different levels of isolation frequency. Consistent with the findings above (Table 2), the combinations that used BA as the second selective culture medium exhibited lower isolation frequencies than those coupled with PA (Table 3). While BB-PA showed the highest (60.0%) frequency of *Campylobacter* isolation, BB-BA exhibited the lowest (2.5%) frequency (Table 3).

**Table 3:** Frequencies of *Campylobacter* isolation from retail raw chicken of four different combinations of Bolton and Preston *Campylobacter*-selective media.^†^

Species	BB-BA	BB-PA	PB-BA	PB-PA
*Campylobacter coli*	0/40 (0.0%)	11/40 (27.5%)	5/40 (12.5%)	1/40 (2.5%)
*Campylobacter jejuni*	1/40 (2.5%)	18/40 (45.0%)	7/40 (17.5%)	12/40 (27.5%)
*C. coli* & *C. jejuni*	0/40 (0.0%)	5/40 (12.5%)	3/40 (7.5%)	1/40 (2.5%)
*Campylobacter*-positive samples	1/40 (2.5%)	24/40 (60.0%)	9/40 (22.5%)	12/40 (30.0%)

† Bolton broth-Bolton agar (BB-BA), Bolton broth-Preston agar (BB-PA), Preston broth-Bolton agar (PB-BA), and Preston broth-Preston agar (PB-PA).

## Discussion

Bacterial isolation from food, environmental, and clinical samples is based on the selective growth and enrichment of the target bacteria using culture media supplemented with antimicrobials to which the target bacteria are intrinsically resistant. If some indigenous bacteria in the background microflora of the sample are resistant to the antimicrobials used in the selective media, the resistant bacteria may compete with target bacteria, affecting the composition of microbiota. This may consequently affect the isolation efficiency, particularly when background microflora outgrows the target bacteria. However, differences in microbiota compositions after the selective enrichment have not been taken into consideration to optimize protocols for bacterial isolation.

In this study, we found that BB markedly increased the proportion of *Escherichia* and reduced that of *Campylobacter* compared to PB (Figure 3). As BB contains three antibiotics (i.e., cefoperazone, vancomycin, and trimethoprim), *E. coli* growing in BB was resistant to the three antibiotics (data not shown). While vancomycin is effective against Gram-positive bacteria, Gram-negative bacteria are usually resistant to vancomycin intrinsically due to the permeability barrier of outer-membrane (Zhou et al., 2015). *E. coli* showed an increasing trend of trimethoprim resistance, and 13.8% of *E. coli* isolates from chicken are resistant to trimethoprim in the United States (Tadesse et al., 2012). In addition, cephalosporin-resistant *E. coli*, such as ESBL- and AmpC-producing *E. coli*, is highly prevalent in poultry (Overdevest et al., 2011; Reich et al., 2013; Olsen et al., 2014). Consistently, in this study, *Escherichia* was predominant in the selective enrichment media (Figure 3A, Table 1). The levels of *E. coli* and *C. jejuni* are originally low on chicken carcasses, which are approximately 0.8 log CFU/ml and 0.02 log CFU/ml in post-chill rinses of chicken carcasses, respectively (Altekruse et al., 2009). However, our study showed that *Escherichia* was highly enriched and became predominant after enrichment with *Campylobacter*-selective media, particularly BB (Figure 3A, Table 1). Furthermore, the high prevalence of *Escherichia* was accompanied by the reduction of *Campylobacter* population (Figure 3B, Table 2).

The different antibiotic selective pressures generated by BB and PB influenced in the formation of unique microbiota structures with the differential levels of *Campylobacter* prevalence in the enrichment cultures. Due to the fecal contamination of poultry carcasses during processing, the composition of microbiota on poultry carcasses is substantially affected by the gut microflora of poultry. Firmicutes is the predominant phylum throughout the chicken intestines from crop to large intestines (Yeoman et al., 2012) and constitutes approximately 50–90% of all taxa in the cecum (Qu et al., 2008; Danzeisen et al., 2011). According to a report from Kim et al. (Kim et al., 2017) on microbiome changes on chicken carcasses, Firmicutes are predominant in all steps of poultry processing. In this study, consistently, Firmicutes, such as *Lactobacillus, Phascolarctobacterium, Coprobacillus*, and *Clostridium_g6*, were detected after BB and PB enrichments (Figure 2A). *Lactobacillus* is frequently detected in the gastrointestinal tracts of chickens and highly abundant (ca. 68%) in the duodenum and ileum (Lu et al., 2003). *Lactobacillus acidophilus* and *Lactobacillus delbrueckii* are dominant in the ileum of chickens aged 3–21 days, and *Lactobacillus salivarius* and *Lactobacillus crispatus* are dominant *Lactobacillus* spp. in chickens aged 28–49 days (Lu et al., 2003); these *Lactobacillus* were isolated by BB-PA (Table 2). In the cecum of chickens, Clostridiaceae and Fusobacterium are dominant (ca. 65 and 14%, respectively) (Lu et al., 2003). *Fusobacterium* is more frequently found in the feces of chickens (Yan et al., 2017), and *Fusobacterium mortiferum* is most abundantly found in chicken feces (Oakley et al., 2013). In our study, fusobacteria were more frequently found in PB-enriched samples, compared with the BB-enriched samples (Figures 2B,C, Table 1), and *F. mortiferum* was isolated in PB-BA (Table 2). The detection of bacterial species that are originated from the gastrointestinal tracts of chickens strongly indicates fecal contamination of chicken carcasses. However, these predominant bacteria in the chicken intestines became less dominant during the enrichment step using the Bolton and Preston selective media.

Our study also highlights the need for the evaluation of bacterial competition in the microbiota of selective enrichment. While a previous report suggested that *Pseudomonas* spp. could support the survival of *C. jejuni* on chicken meat (Hilbert et al., 2010), our results showed that *Pseudomonas* was overrepresented in the BB-enriched samples with a low abundance of *Campylobacter* compared to the PB-enriched samples (Figure 2B). In addition, it has been reported that several *Lactobacillus* strains showed antagonistic activities against *C. jejuni* (Lehri et al., 2017), suggesting that higher abundance of *Lactobacillus* in the BB-enriched samples might affect the growth of *Campylobacter* in the BB media. Further studies are needed to elucidate the dynamics of competitive bacterial growth under selective enrichment conditions.

Interestingly, the application of the secondary selective culture generated completely different frequencies of *Campylobacter* isolation. In addition to the different capability of the selective media in enriching *Campylobacter* (Figure 3), the sequence of combining the selective media substantially affected the isolation frequency. Using the same selective culture media (Bolton and Preston), BB-PA exhibited significantly higher frequencies of *Campylobacter* isolation than PB-BA by 5.9-fold (Table 3). The ISO protocol for *Campylobacter* detection employs the enrichment with BB and subsequent culture on mCCDA that is supplemented with cefoperazone, a third-generation cephalosporin, which is one of the three antibiotics in the Bolton selective supplement. Based on the antibiotics used in the protocol, the combination of BB and mCCDA would generate a selective pressure similar to that of the BB-BA combination. To improve the isolation frequency, the revised version of ISO includes the enrichment with Preston broth and the following culture on mCCDA (ISO, 2015); this would possibly be similar to the PB-BA combination. In our study, the detection frequency of PB-BA (22.5%) was higher than that of BB-BA (2.5%), whereas the best isolation frequency was achieved in the BB-PA combination (Table 3).

The prevalence of *Campylobacter* in poultry is usually high in many countries; however, the prevalence level often varies depending on the country. Accurate determination of a baseline of *Campylobacter* contamination of foods is highly important, since the baseline level may affect the results of a risk assessment of *Campylobacter* contamination of poultry (Habib et al., 2008) and food safety policies pertaining to the control of *Campylobacter* in the food supply system. Although NGS is a powerful tool to investigate microbiota, it has never been used to investigate microbiota compositions during the selective culture of bacteria, although the microbiota compositions may significantly impact the frequencies of bacterial isolation. Aiming to improve the frequencies of *Campylobacter* isolation from poultry carcasses, this study provided a novel insight into how an NGS-based microbiota analysis can be employed to optimize the protocols for *Campylobacter* isolation. The same approach can be applied to the development of isolation protocols for other bacteria.

## Author Contributions

SC, SR, and BJ designed the study. JK, HS, HP, HJ, and JHK performed the experiments. JK, HS, SR, and BJ analyzed the data. JK, HS, and BJ wrote the manuscript. JK, HS, SC, SR, and BJ reviewed the manuscript.

### Conflict of Interest Statement

The authors declare that the research was conducted in the absence of any commercial or financial relationships that could be construed as a potential conflict of interest.
