# Supplementary material for: Microbiota Analysis for the Optimization of Campylobacter Isolation From Chicken Carcasses Using Selective Media
Source: Front Microbiol. 2019 Jun 21;10:1381. doi: 10.3389/fmicb.2019.01381 (PMC6598470; doi:10.3389/fmicb.2019.01381)
Supplement: Supplementary file 1 [file Data_Sheet_1.PDF]

Table S1. Summary of analyzed sequence information

**Selective medium**	**Bolton**	**Preston**	**Total**
Number of samples	20	20	40
Total number of sequences	830,539	911,056	1,741,595
Mean number of sequences ± SD	41,527 ± 10,787	45,553 ± 12,490	43,540 ± 11,842
Total number of sequences yielding OTUs	780,878	851,474	1,632,352
Mean number of sequences yielding OTUs ± SD	39,044 ± 10,839	42,574 ± 12,997	40,809 ± 12,096
Number of observed OTU types	2,853	3,078	4,508
Mean number of observed OTU types	362 ± 223	348 ± 181	355 ± 203

Table S2. Relative abundance of bacterial taxa (top 27 taxonomies) in enriched-chicken samples by selective media

Bacterial taxa	Bolton broth		Preston broth	
	Average (%)	SD (%)	Average (%)	SD (%)
Unassigned	0.010494	0.007518	0.017376	0.03029
Pseudomonas[a]	0.066993	0.107706	0.018384	0.044282
Acinetobacter[a]	0.01428	0.024536	0.002345	0.005418
Yersinia	0.003936	0.008576	0.000875	0.002104
Serratia	0.011017	0.016703	0.003588	0.008121
Salmonella	0.000116	0.000228	0.010921	0.043578
Proteusa[b]	0.001982	0.003262	0.051391	0.113173
Enterobacteriaceae[*]	0.008456	0.015365	0.019549	0.035497
Klebsiella	0.001407	0.003676	0.030065	0.080348
Hafnia	0.001592	0.003657	0.006955	0.015623
Escherichia[a]	0.706185	0.205485	0.435565	0.296455
Enterobacter[b]	0.001137	0.002599	0.006445	0.012079
Buttiauxella	0.002498	0.00715	0.000545	0.000811
Shewanella	0.01823	0.031937	0.010784	0.020126
Aeromonas	0.01322	0.032661	0.029992	0.083564
Campylobacter	0.000364	0.00065	0.008857	0.033308
Fusobacterium[b]	0.024574	0.101321	0.248409	0.322747
Megamonas	0.045957	0.079532	0.006572	0.024253
Phascolarctobacterium[a]	0.012729	0.030239	0.000315	0.001248
Erysipelothrix[b]	0	0	0.011613	0.038189
Coprobacillus[b]	0.000047	0.000109	0.026078	0.070242
Clostridium_g6[b]	0.00001	0.000032	0.018655	0.037892
Clostridium	0.000713	0.001711	0.003041	0.01182
Lactococcus	0.003121	0.007287	0.008418	0.0152
Lactobacillus[a]	0.025155	0.033968	0.002922	0.011788
Carnobacterium	0.003578	0.008317	0.000281	0.00087
Brochothrix	0.001611	0.006086	0.002696	0.005547
Bacteroides[a]	0.003504	0.00653	0.000136	0.000323

[*]Unidentified genus in family level; [a], Over represented major taxa in Bolton group
[b], Over represented major taxa in Preston group

Table S3. Primers used in this study

Gene	Primer	Sequence (5′-3′)	Reference
Campy-16S rDNA	C412F	GGATGACACTTTTCGGAGC	Yamazaki-Matsune et al. 2007
	C1228R	CATTGTAGCACGTGTGTC	
C. jejuni-cj0414	C-1	CAAATAAAGTTAGAGGTAGAATGT	Yamazaki-Matsune et al. 2007
	C-3	CCATAAGCACTAGCTAGCTGAT	
C. jejuni-hipO	hipO_F	GCAAAATCCACAGCTTCATCGT	This study
	hipO_R	GGAAGGGGTGGTCATGGAAG	
C. coli-Ask	CC18F	GGTATGATTTCTACAAAGCGAG	Yamazaki-Matsune et al. 2007
	CC519R	ATAAAAGACTATCGTCGCGTG	
C. lari-glyA	CLF	TAGAGAGATAGCAAAAGAGA	Yamazaki-Matsune et al. 2007
	CLR	TACACATAATAATCCCACCC	
Universal-16S rDNA	341F	CCTACGGGNGGCWGCAG	Eiler et al. 2013
	805R	GGACTACHVGGGTWTCTAAT	

References

1. Yamazaki-Matsune, W., Taguchi, M., Seto, K., Kawahara, R., Kawatsu, K., Kumeda, Y., Kitazato, M., Nukina, M., Misawa, N., and Tsukamoto, T. (2007). Development of a multiplex PCR assay for identification of *Campylobacter coli*, *Campylobacter fetus*, *Campylobacter hyointestinalis* subsp. *hyointestinalis*, *Campylobacter jejuni*, *Campylobacter lari* and *Campylobacter upsaliensis*. *J. Med. Microbiol.* 56,1467-1473.
2. Eiler, A., Drakare, S., Bertilsson, S., Pernthaler, J., Peura, S., Rofner, C., Simek, K., Yang, Y, Znachor P, and Lindstrom ES. (2013). Unveiling distribution patterns of freshwater phytoplankton by a next generation sequencing based approach. *PLoS One* 8, e53516.

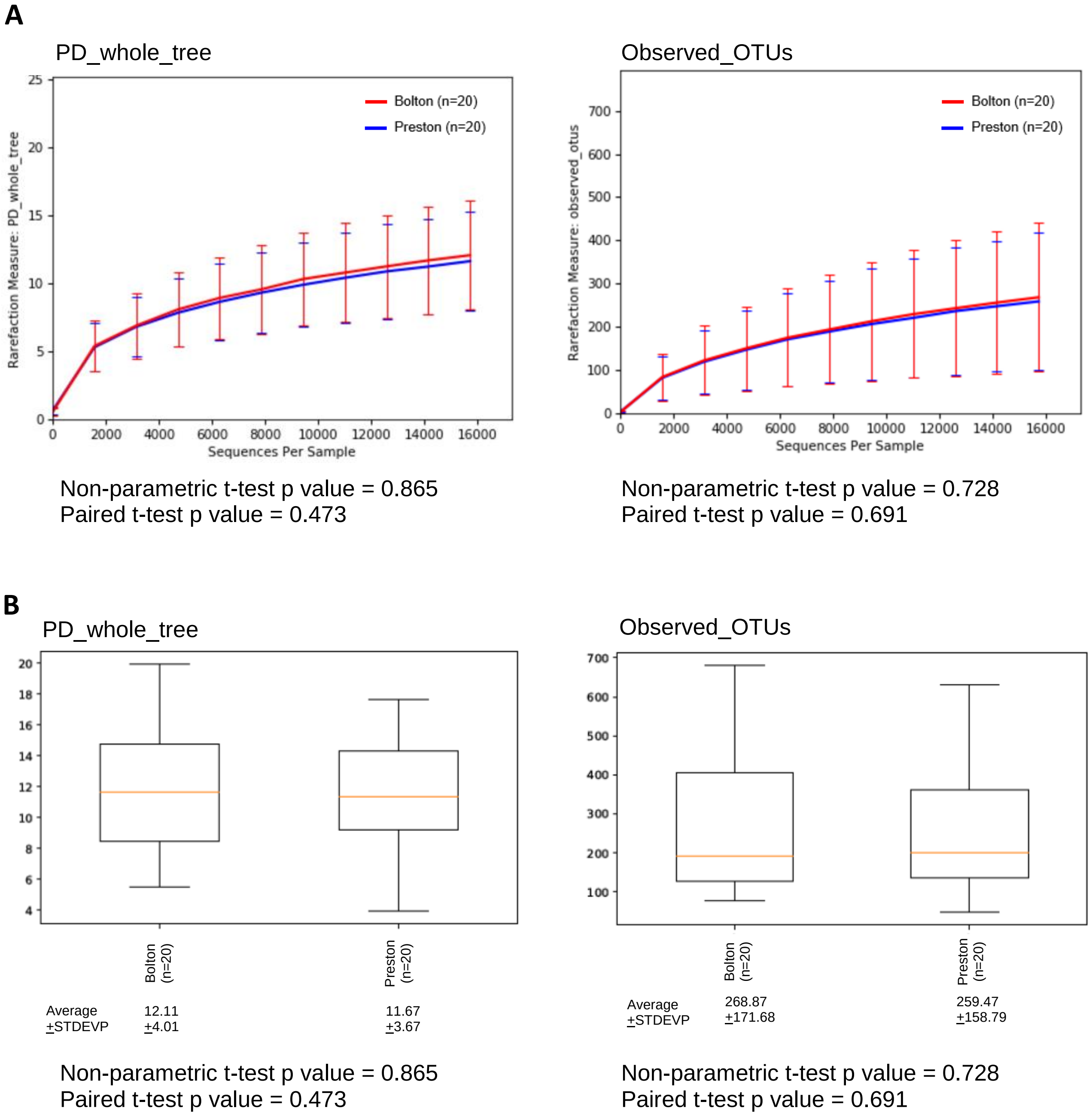

FIG. S1. Bacterial alpha diversity of the microbiota in chicken carcasses after enrichment with the Bolton and Preston *Campylobacter*-selective media. (A) Rarefaction plots. **(B)** Box plots. All communities were rarefied up to 15,030 reads per sample to calculate bacterial diversity. The non-parametric *p* values were calculated using 100,000 Monte Carlo permutation.

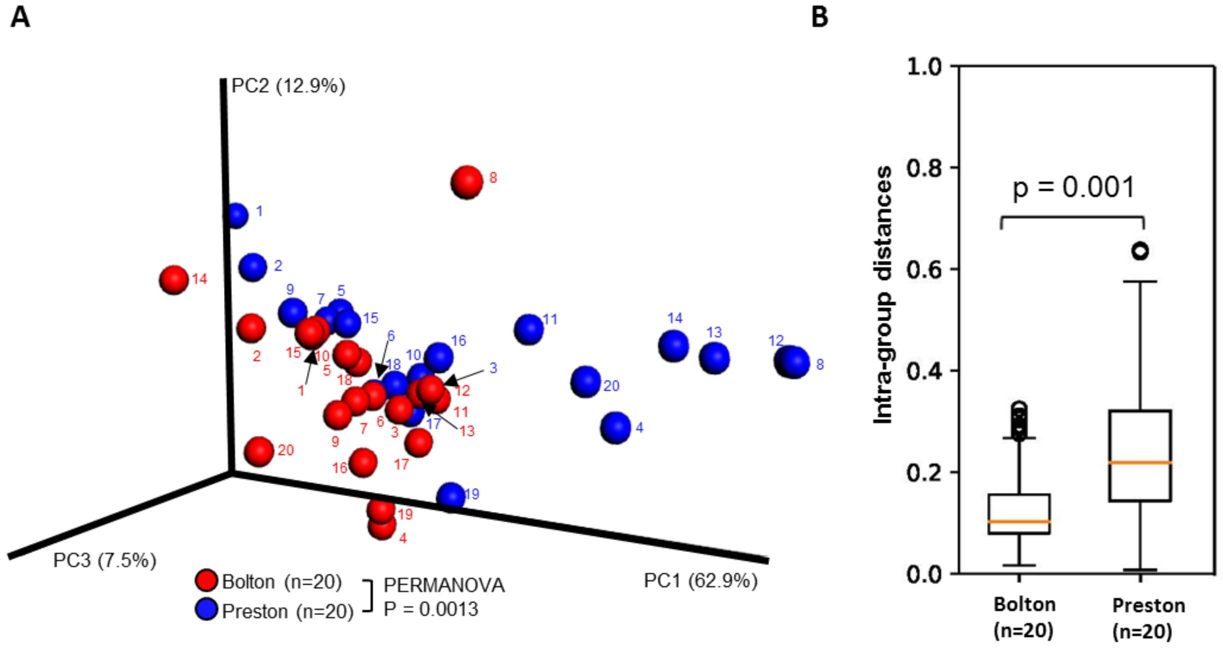

FIG. S2. Bacterial beta diversity of the microbiota in chicken carcasses after enrichment with the Bolton and Preston *Campylobacter*-selective media. (A) Principal Coordinate Analysis (PCoA) plot of bacterial communities in enriched-chicken samples by selective media. Weighted UniFrac distances were used to evaluate diversity between samples and PERMANOVA was used to test dissimilarity of bacterial structure. **(B)** Box plot of intra-group distances. The non-parametric *p* values were calculated using 100,000 Monte Carlo permutation.
